# The Safety and Feasibility of Laparoscopic Gastrectomy after Neoadjuvant Chemotherapy for Locally Advanced Gastric Cancer

**DOI:** 10.1155/2022/9511066

**Published:** 2022-05-31

**Authors:** Rui Ge, Kai Liu, Weihan Zhang, Kun Yang, Xiaolong Chen, Linyong Zhao, Zongguang Zhou, Jiankun Hu

**Affiliations:** ^1^Department of Gastrointestinal Surgery and Laboratory of Gastric Cancer, State Key Laboratory of Biotherapy, West China Hospital, Sichuan University, and Collaborative Innovation Center for Biotherapy, Chengdu, China; ^2^Department of Gastrointestinal Surgery and Laboratory of Digestive Surgery, State Key Laboratory of Biotherapy, West China Hospital, Collaborative Innovation Center for Biotherapy, Sichuan University, Chengdu, China

## Abstract

**Background:**

Neoadjuvant chemotherapy is incrementally applied to remedy locally advanced gastric cancer. However, NACT also enhances the difficulty of laparoscopic lymph node dissection. The objective of our study was to evaluate the safety and feasibility of laparoscopic gastrectomy for locally advanced gastric cancer after neoadjuvant chemotherapy.

**Methods:**

From July 2017 to December 2019, 153 patients who received neoadjuvant chemotherapy and underwent the subsequent surgical procedure were retrospectively enrolled and analyzed in the Gastrointestinal Surgery Department of West China Hospital. According to surgical methods, all the patients were sectionalized into two groups: laparoscopic assistant gastrectomy (LAG, 77 patients) and traditional open gastrectomy (OG, 76 patients). The demographic parameters, preoperative, surgical, pathological, and neoadjuvant chemotherapy features were compared between the two groups.

**Results:**

A total of 153 patients accepted neoadjuvant chemotherapy and surgical resection in our study. There was no statistically significant difference in demographic parameters and preoperative and neoadjuvant chemotherapy characteristics between the two groups. The LAG group illustrated less intraoperative blood loss (91.1 ± 53.1 ml vs. 125.7 ± 116.9 ml, *p*=0.010) and shorter postoperative hospital stays (7.9 ± 2.1 days vs. 125.7 ± 116.9 days, *p*=0.009), when compared to the OG group. Moreover, there was no disparity with respect to operative duration, number of harvested lymph nodes, and postoperative complication rates between the two groups. When considering the Clavien–Dindo classification, no statistically significant difference was indicated in all stratifications with regard to postoperative complications.

**Conclusion:**

Laparoscopic gastrectomy for locally advanced gastric cancer after neoadjuvant chemotherapy is safe and feasible without increasing postoperative adverse events.

## 1. Introduction

Locoregionally advanced gastric cancer (LAGC) poses a rigorous challenge to the treatment and prognosis of gastric cancer, especially in China [1, 2]. Multidisciplinary treatment based on an accurate clinical stage is mainstream in recent years, and curative surgery and neoadjuvant chemotherapies (NACTs) are crucial procedures in the therapeutic process of LAGC [3]. The MAGIC research initially affirmed the benefit of NACT for gastric cancer in the Western population, and the FNCLCC trial and Chinese RESOLVE trial also testify the salutary effect of NACT for gastric cancer [4–6]. The conceivable advantages of NACT involve tumor downstaging, preferable option of chemotherapy regimen, and furtive micrometastasis obliteration [6].

During the past 2 decades, the surgical procedure for LAGC has been transformed from traditional open gastrectomy to minimally invasive operation. Laparoscopic gastrectomy has been widely accepted and become a standard treatment for early gastric cancer [7–9]. In addition, 3 distinguished open-label, randomized controlled (JLSSG0901 in Japan, KLASS-02 in South Korea, and CLASS-01 in China) trials extended the indications of laparoscopic distal gastrectomy in LAGC [10–12]. Recently, the safety and reliability of laparoscopic total gastrectomy were also demonstrated by CLASS-02 and CLASS-04 trials in China [13, 14]. The available pieces of evidence indicate that laparoscopic gastrectomy could obtain comparable short-term and long-term results as conventional open gastrectomy without increment of supererogatory risk even for LAGC.

As we all know that NACT is able to improve the prognosis of LAGC, however, NACT is also detriment normal tissue and anatomic plane, and the tissue edema and fibrosis propose many troubles to laparoscopic surgical technique. Besides, the cytotoxicity of NACT may also have an impact on the perioperative recovery [15]. With regard to the indication of laparoscopic gastrectomy for patients after NACT, 2 RCTs have testified the postoperative safety and adjuvant chemotherapy tolerance compared with open surgery [15, 16]. In addition, there are 3 retrospective studies that also manifested the feasibility and non-inferiority of laparoscopic gastrectomy [17–19]. Although more clinical pieces of evidence with larger sample RCT trials concerning this hotspot are still warranted, laparoscopic gastrectomy for LAGC after NACT is an alternative approach for experienced surgeons. There is no doubt that the criterion for the recruitment of patients who underwent laparoscopic gastrectomy after NACT is supposed to be more meticulous and response evaluation of NACT should be more prudent and recognized. The purpose of this research was to further evaluate the safety and feasibility of laparoscopic gastrectomy in LAGC patients after NACT.

## 2. Materials and Methods

### 2.1. Patients

**Figure 1:**
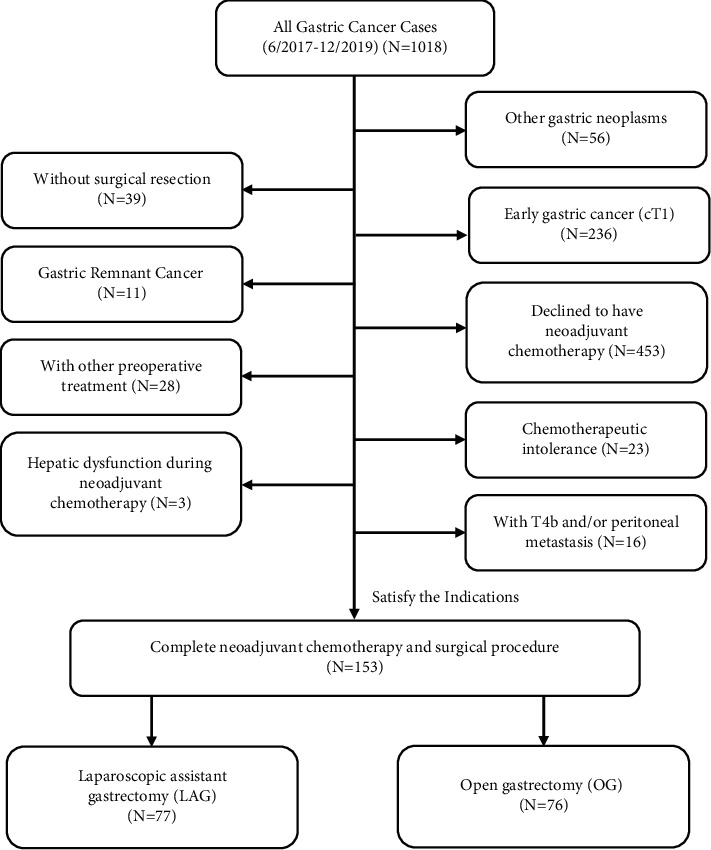
Flow diagram of patient enrollment in this study.

A total of 153 patients with LAGC after NACT were enrolled in a gastric cancer professional group in the Gastrointestinal Surgery Department, West China Hospital, from July 2017 to December 2019. Patients who were evaluated with early stage (cT1), with distant metastasis, without surgical resection, other gastric neoplasms, refused NACT, received other preoperative treatment, completed less than 2 cycles of NACT, chemotherapeutic intolerance, or tumor exacerbation without resection were excluded from this study. As a consequence, 153 cases were left in the final analysis: 77 patients in the laparoscopic assistant gastrectomy (LAG) group and 76 patients in the open gastrectomy (OG) group (Figure 1). All the patients were assigned individual treatment strategy in terms of their preoperative staging, and the chemotherapeutic effect was mainly evaluated by high-quality computed tomography (CT) scan, gastroscope, and gastrointestinal ultrasonography (GUS). The definitions including Borrmann types and clinicopathological features were chiefly in line with the 14th edition of the Japanese Classification of Gastric Carcinoma by JGCA [20]. All the tumor TNM staging included clinical stage, and pathological stage after NACT was according to the 8th edition of the AJCC cancer staging manual [21].

### 2.2. Neoadjuvant Chemotherapy (NACT)

Before NACT, there were 91.5% of patients in our study conducted with “four-step” laparoscopic exploration (LE) to evaluate the intraperitoneal metastasis and acquire an accurate clinical stage [22]. For patients with cT2 or more advanced without explicit distant metastasis or peritoneal dissemination, the NACT was introduced to the patients for consideration. Patients received 2–6 cycles of NACT before the surgical procedure. The regimens of NACT include XELOX regimen: oxaliplatin, 150 mg/m^2^, by intravenous infusion, on day 1 of every 3 weeks, and capecitabine, 1000 mg/m^2^, orally, twice daily from day 1 to day 14 every 3 weeks; SOX regimen: intravenous oxaliplatin 130 mg/m^2^ on day 1, plus oral S-1 40 mg/m^2^ twice daily on days 1 to 14 of each cycle, every 21 days; FOLFOX regimen: oxaliplatin 85 mg/m^2^ and leucovorin 400 mg/m^2^ were administered as an intravenous infusion, followed by a 5-FU bolus of 400 mg/m^2^ and 5-fluorouracil (5-FU) 2400 mg/m^2^ as a 46-hour continuous infusion every 14 days; and FLOT regimen: intravenous 5-FU 2600 mg/m^2^ via peripherally inserted central catheter (PICC) continued for 24h on day 1, intravenous leucovorin 200 mg/m^2^, intravenous oxaliplatin 85 mg/m^2^, and intravenous docetaxel 50 mg/m^2^, and the next cycle was repeated on the 15th day. The indication of NACT in our study was mainly cT3/T4N+, and few cT2N0 cases were also enrolled. Adverse effects were recorded according to the National Cancer Institute Common Terminology Criteria for Adverse Events (CTCAE 4.0). Drug dose or timing was adjusted for patients with grade 3 and above adverse effects. Chemotherapeutic dosage adjustment or termination was intervened once patients were subjected to severe or fatal adverse events. The clinical response of NACT was assessed by both radiologists and surgeons after a comparison of pre- and post-chemotherapy radiological images following the guideline of Response Evaluation Criteria in Solid Tumors (RECIST) [23]. Within 6–7 weeks after completing the last cycle, the surgical approach was subsequently evaluated and conducted.

### 2.3. Surgical Treatment

Based on the results of previous prospective RCT trials and clinical pieces of evidence [11, 12, 14, 15], the indication for laparoscopic gastrectomy for patients after NACT was only considered as follows: 1. the clinical stage was ycT0-4aN0/+M0H0P0Cy0; 2. patients with favourable clinical response of NACT including complete response and/or partial response; 3. patients without previous abdominal operation; 4. patients with sufficient tolerance for laparoscopic surgery; and 5. patients without suspicious metastasis.

In our study, surgical schemes for gastric cancer after NACT were in accordance with Japanese gastric cancer treatment guidelines in 2014 (4th edition) and 2018 (5th edition) [8, 24]. A normative gastrectomy with D2 or D2 plus lymphadenectomy (involving nos. 1/3/4sb/4d/5/6/7/8a/9/11p and 12a were dissected in distal gastrectomy; no. 1/2/3/4sa/4sb/4d/5/6/7/8a/9/11p/11d/12a was dissected in total gastrectomy) was performed in both the LAG and OG groups. The resection range was determined by the tumor site and diameter. Frozen biopsy was routinely conducted to ensure the safety of incisal margins. The reconstruction method for distal gastrectomy included standard Billroth I, Billroth II, and Roux-en-Y anastomosis, depending on the size of gastric remnant and the butcher physician's preference. Roux-en-Y anastomosis was conducted after total gastrectomy. All the operations were performed by one experienced surgeon. An experienced surgeon was specialized in the separation of lymph nodes on the biopsy for every operation. The postoperative complication was defined as adverse events occurring within 30 days after surgery, and the severity was identified by the Clavien–Dindo classification system [25, 26].

### 2.4. Statistical Processing

The categorical variables were shown as number and percentage (%), and continuous variables were described as mean and standard deviation. All the variables underwent a normality test and homogeneity test of variance. In our study, all the variables were demonstrated with skewed distribution and nonparametric tests were conducted. The Mann–Whitney *U* test was used to analyze continuous variables, whereas the Wilcoxon rank-sum test was used for ordered categorical variables and the chi-square test and Fisher's exact test were applied for unordered categorical variables. The above evaluation was performed by *R* software (Version 4.0.1. https://www.r-project.org/). A *p* value <0.05 (2-tailed) was defined to be statistically significant.

## 3. Results

### 3.1. Patients and Clinical Characteristics

We applied the technique of LASPLND to LAGC after NACT since July 2017. From July 2017 to December 2019, 153 LAGC patients finally completed NACT and surgical procedure in a professional gastric cancer group at West China Hospital. The flow diagram of evaluation is shown in Figure 1. There were 77 (50.3%) patients in the LAG group and 76 (49.7%) patients in the OG group. There were 53 (66.8%) male and 24 (31.2%) female patients in the LAG group and 59 (77.6%) male and 17 (22.4%) female patients in the OG group (*p*=0.295). There was no significant difference in the age (LAG: 60.4 ± 9.4 vs. OG: 59.3 ± 10.6, *p*=0.675), body mass index (BMI) (LAG: 23.0 ± 3.2 vs. OG: 23.7 ± 2.9, *p*=0.188), preoperative hemoglobin (LAG: 123.9 ± 17.3 vs. OG: 120.9 ± 21.4, *p*=0.487), and albumin (LAG: 42.3 ± 2.9 vs. OG: 42.0 ± 3.2, *p*=0.434) between the LAG and OG groups. In the LAG group, patients have a smaller tumor size (3.6 ± 1.7 vs. 4.5 ± 2.6, *p*=**0.038**) when compared to the OG group. No significant discrepancy was detected in tumor longitudinal location (*p*=0.329), Borrmann types (*p*=0.233), and yc clinical stage: ycT (*p*=0.722), ycN (*p*=0.955), and ycTNM (*p*=0.939). The basic clinicopathological features of the two groups are shown in Table 1.

### 3.2. Neoadjuvant Chemotherapy Characteristics

The comparison of NACT characteristics is summarized in Table 2. The NACT characteristics were well balanced between the two groups: there was no statistically significant disparity in ANCT regimens (*p*=0.271), completed cycle of NACT (*p*=0.0.78), proportion of downstage (LAG: 76.6% vs. OG: 69.7%, *p*=0.336), and the interval duration between NACT completed and surgical procedure (LAG: 6.3 ± 1.7 vs. OG: 6.7 ± 2.0, *p*=0.336). The rate of clinical response of the LAG group is as follows: complete response (CR): 6.5%, partial response (PR): 61.0%, stable disease (SD): 31.2%, and progressive disease (PD): 1.3%, and that of the OG group is as follows: CR: 2.6%, PR: 72.4%, SD: 40.8%, PD: 3.9%, and no significant difference between the two groups (*p*=0.330). According to the CTCAE 4.0, the incidence of grade 2–4 adverse effect after NACT was also similar between the two groups (LAG: 22.1% vs. OG: 26.3%, *p*=0.327).

### 3.3. Surgical and Pathological Characteristics

The surgical and pathological parameters of two groups are indicated in Table 3. In the LAG group, there were a higher proportion of distal gastrectomy (49.4% vs. 27.6%, *p*=0.010) and significantly less intraoperative blood loss (91.1 ± 53.1 vs. 125.7 ± 116.9, *p*=0.024) than the OG group. There was no statistically significant proportion of LE (*p*=0.980), surgical radicalness (*p* > 0.999), range of lymphadenectomy (*p* > 0.999), harvested lymph node number (*p*=0.165), tumor differentiation (*p*=0.527), proportion of signet-ring cell carcinoma (*p*=0.469), Lauren type (*p*=0.431), tumor regression grade (*p*=0.269), ypT stage (*p*=0.915), ypN stage (*p*=0.531), and ypTNM stage (*p*=0.354).

### 3.4. Lymph Node Dissection

The effectivity and safety are reflected in Tables 3 and 4. The LAG achieved considerable outcomes on the number of total examined lymph nodes (41.6 ± 12.4 vs. 40.0 ± 14.3, *p*=0.165) when compared to the OG group. There was also no difference in the rate of lymphatic leakage (*p*=0.497), pancreatic fistula (*p*=0.497), anastomotic leakage (*p*=0.497), and intraperitoneal hemorrhage (*p*=0.497). There was no intraabdominal infection or surgical mortality observed in both the two groups.

### 3.5. Postoperative Hospital Stays and Complications

The postoperative stays and rate of complication are depicted in Table 4. For the LAG groups, patients have a significantly shorter postoperative hospital stays than that in the OG group (7.9 ± 2.1 vs. 9.3 ± 5.8, *p*=**0.009**). When concerned with the rate of postoperative complication: no statistical discrepancy was indicated between the two groups (LAG: 18.2% vs. OG: 30.3%, *p*=0.120), and even LAG showed an advantage in this respect. After the Clavien–Dindo classification, there was no obvious difference in grade I (*p* > 0.999), grade II (*p*=0.207), and grade IIIb (*p* > 0.999). LAG might indicate lower proportion of grade IIIa complications (0.0% vs. 5.3%, *p*=0.058) and unplanned readmission (1.3% vs. 6.6%, *p*=0.116); however, no statistical significance was notarized.

## 4. Discussion

In recent years, the short- and long-term effects of neoadjuvant chemotherapy on advanced gastric cancer were accredited and widely recommended [4–7]. Meanwhile, the role of laparoscopic distal and total gastrectomy in locally advanced gastric cancer was also affirmed by many convictive clinical trials [10–12, 14, 27]. The NCCN guideline also recommends that NACT should be a preferred selection for gastric patients with T2 or more stages [28]. The safety and validity of LAG after NACT were also detected by several previous research studies [15–19]. Based on the previous experience of laparoscopic lymph node dissection, we also found that the oncological outcomes of LAG could be comparable to OG according to the number of total harvested lymph nodes, suprapancreatic lymph nodes, postoperative morbidity, and mortality. Furthermore, the technique of LASPLND might bring less blood loss without protracting the surgical duration. For the LAG, the postoperative recovery also preceded traditional open surgery. Therefore, the LAG was safe for LAGC after NACT.

By the means of NACT, a fairly proportion of LAGC patients might obtain a better prognosis. The popularization of NACT and minimally invasive is an inevitable tendency in the treatment of LAGC. However, NACT was a systemic treatment and consequentially caused an adverse impact on the surgical operation and postoperative recovery. To furthest reduce the adverse impact of NACT, a technical and programmed procedure was demanded imminently. Although the standard procedure of laparoscopic lymph node dissection had been reported [29, 30], the feasibility of these procedures in LAGC after NACT was still inconclusive. We found that patients after NACT were more prone to suffer from concomitant tissue severe edema, fibrosis, and effusion, and the normal and customary anatomical layer was also defunct, especially for the suprapancreatic area. We also established a gross tissue response (GTR) system to predict the risk of a difficult operation after NACT and postoperative complications [31]. Higher GTR sore was associated with surgical trauma and postoperative complications. The dissection of interstitial tissues around pancreas was the foundation of gastric cancer surgery; however, peripancreatic texture after NACT was more prone to tissue laceration and capillary bleeding during the surgical process. Compared with surgery without NACT, another conspicuous distinction was tissue response to NACT that compressed the peripancreatic space and made it tougher to build the manipulative tunnels along vessels. Undoubtedly, these adverse factors increased the surgical trauma and potential accidents and summarized the technique contrapose to the abovementioned drawbacks.

We summarized the advantage of laparoscopic lymph node dissection for LAGC after NACT such as guaranteeing the continuity convention of dissection, avoiding unwanted injury, seeking manipulative space, and coping with effusion and bleeding of this area effectively. The advantage of 4K laparoscopy in our study included the following: in the first place, the 4K laparoscopy enhanced the discrimination of anatomical layer, blood, and lymphatic vessel and the demarcation between lymph nodes and pancreas and also improved the resolution of tiny lymph nodes. These were all conducive to elaborate dissection, reduce the rate of postoperative pancreatitis, pancreatic fistula, lymphatic and chylous leakage, and avert lymph node residual. On the other hand, the intelligent adjustment of aspirator and camera provided a clear surgical field and overlook view to distinguish the splenic artery and vein and lymph nodes behind the pancreas. This is an outstanding technique to prevent injury of pancreas and splenic vessels. Furthermore, the sequence of clockwise and modularized lymphadenectomy could ensure a monodirectional operation, and the former step prepared the manipulative room and scope for the next step until all the procedures were finished [32].

The blood loss and postoperative complication incidence were also in accordance with our results. For patients after NACT, since the regression of lymph nodes and the compact structure, the harvested lymph nodes might be reductive. Compared with other studies without NACT, our LAG group still could harvest more than 40 lymph nodes and maintain sufficient lymphadenectomy for LAGC. These also illustrated that the procedures introduced in our study had absolute qualification on laparoscopic dissection and deserve to be popularized. An RCT trial from China reported the safety of laparoscopic distal gastrectomy for LAGC after NACT, and the mean number of examined lymph nodes in the laparoscopic group was 31 with 20% postoperative complication [15]. The main outcome was also comparative with us. Another European RCT concentrated on the safety and feasibility of minimally invasive total gastrectomy for LAGC after NACT indicated similar harvested lymph nodes (mean number: 41.7) and a higher rate of perioperative complication (34.0%) [16].

Seldom limitations consisted in our study are as follows: firstly, this was retrospective with an inadequate sample size that might have potential confounding factors and affect the final results. Secondly, there are four regimens included in our study, and the concordance of the impact on the surgery might be impaired. Thirdly, the regimen of the “LE-NACT-surgical procedure” was relatively costly, the surgery is postponed, and many patients feel troublesome and are afraid of tumor progress. Quite a number of patients with LAGC refused NACT, even after sufficient explanation by us, and lead to partial patients who satisfy the indications of NACT that were not affiliated in this cohort.

## 5. Conclusions

Laparoscopic gastrectomy acquired considerable effects without increasing postoperative adverse events when compared with open gastrectomy for locally advanced gastric cancer after neoadjuvant chemotherapy.

## Figures and Tables

**Table 1:** Baseline, demographics, and clinical features of cases in this study.

Demographics and clinical features	LAG	OG	*p* value
	*N* = 77 (%)	*N* = 76 (%)	
Age (years)	60.4 ± 9.4	59.3 ± 10.6	0.675
Gender			0.295
Male	53 (66.8)	59 (77.6)	
Female	24 (31.2)	17 (22.4)	
BMI (kg/m^2^)	23.0 ± 3.2	23.7 ± 2.9	0.188
Hemoglobin (g/L)	123.9 ± 17.3	120.9 ± 21.4	0.487
Albumin (g/L)	42.3 ± 2.9	42.0 ± 3.2	0.434
Tumor size	3.6 ± 1.7	4.5 ± 2.6	**0.038**
Longitudinal location			0.329
Esophagogastric junction	17 (22.1)	25 (32.9)	
Fundus	14 (18.2)	16 (21.1)	
Corpus	17 (22.1)	12 (15.8)	
Antrum	29 (37.7)	22 (28.9)	
The whole stomach	0 (0)	1 (1.3)	
Borrmann type			0.233
Type 0	2 (2.6)	2 (2.6)	
Type I	0 (0)	4 (5.3)	
Type II	21 (27.3)	14 (18.4)	
Type III	48 (63.2)	48 (63.2)	
Type IV	6 (7.8)	8 (10.5)	
ycT stage^*∗*^			0.722
T2	7 (9.1)	7 (9.2)	
T3	23 (29.9)	17 (22.4)	
T4a	42 (54.5)	45 (59.2)	
T4b	5 (6.5)	7 (9.2)	
ycN stage^*∗*^			0.955
N0	7 (9.1)	7 (9.2)	
N1	35 (45.5)	32 (42.1)	
N2	26 (33.8)	26 (34.2)	
N3	9 (11.7)	11 (14.5)	
ycTNM stage^*∗*^			0.939
IIA	7 (9.1)	7 (9.2)	
IIB	7 (9.1)	7 (9.2)	
III	58 (75.3)	55 (72.4)	
IVA	5 (6.5)	7 (9.2)	

BMI : body mass index; ^*∗*^the yc stages were indicated by preoperative CT scan after neoadjuvant chemotherapy, and all staging was based on the eighth edition of the AJCC Cancer Staging Manual by the American Joint Committee on Cancer and International Union Against Cancer.

**Table 2:** Neoadjuvant chemotherapy characteristics of cases in this study.

Neoadjuvant chemotherapy characteristics	LAG	OG	*p* value
	*N* = 77 (%)	*N* = 76 (%)	
Regimen of NACT			0.271
XELOX	70 (90.9)	63 (82.9)	
FOLFOX	2 (2.6)	5 (6.6)	
SOX	5 (6.5)	5 (6.6)	
FLOT	0 (0.0)	3 (3.9)	
Cycles completed			0.078
2 cycles	5 (6.5)	10 (13.2)	
3 cycles	67 (87.0)	55 (72.4)	
4 cycles	5 (6.5)	11 (14.5)	
Clinical response per RECIST criteria			0.330
Complete response	5 (6.5)	2 (2.6)	
Partial response	47 (61.0)	40 (52.6)	
Stable disease	24 (31.2)	31 (40.8)	
Progressive disease	1 (1.3)	3 (3.9)	
Tumor downstage			0.336
Yes	59 (76.6)	53 (69.7)	
No	18 (23.4)	23 (30.3)	
Chemotherapy-surgery interval time (weeks)	6.3 ± 1.7	6.7 ± 2.0	0.327
Grade 2-4 adverse effects of NACT^#^	17 (22.1)	20 (26.3)	0.540
WBC decrease	12 (15.6)	13 (17.1)	0.799
Platelet decrease	10 (13.0)	11 (14.5)	0.789
Neutrophil decrease	13 (16.9)	14 (18.4)	0.803
Anemia	3 (3.9)	4 (5.3)	0.686
Hepatic dysfunction	1 (1.3)	5 (6.6)	0.116
Nausea or vomiting	1 (1.3)	2 (2.6)	0.620
Diarrhea	1 (1.3)	0 (0.0)	>0.999
Itching	2 (2.6)	3 (3.9)	0.681
Asitia	2 (2.6)	2 (2.6)	>0.999
Fatigue	1 (1.3)	3 (3.9)	0.363
Neurotoxic effect	1 (1.3)	0 (0.0)	>0.999

LAG: laparoscopy-assisted gastrectomy; OG: open gastrectomy; NACT: neoadjuvant chemotherapy; WBC: white blood cell. ^#^Adverse effects were recorded according to the National Cancer Institute Common Terminology Criteria for Adverse Events (CTCAE 4.0), and one patient can have more than 1 adverse effects.

**Table 3:** Surgical and pathological characteristics of population in this study.

Surgical and pathological features	LAG	OG	*p* value
	*N* = 77 (%)	*N* = 76 (%)	
Laparoscopic exploration before NACT			0.980
Yes	71 (92.2)	69 (90.8)	
No	6 (7.8)	7 (9.2)	
Surgical radicalness			>0.999
R0	75 (97.4)	74 (97.4)	
R1	2 (2.6)	2 (2.6)	
Resection range			**0.010**
Distal	38 (49.4)	21 (27.6)	
Total	39 (50.6)	55 (72.4)	
Range of dissection			>0.999
D2	73 (94.8)	73 (96.1)	
D2+	4 (5.2)	3 (3.9)	
Operative duration	302.4 ± 49.9	296.6 ± 50.2	0.449
Intraoperative blood loss	91.1 ± 53.1	125.7 ± 116.9	**0.024**
Total no. of lymph nodes dissected	41.6 ± 12.4	40.0 ± 14.3	0.165
No. of lymph node metastasis	3.0 ± 4.8	4.6 ± 6.4	0.290
Differentiation			0.527
Well	0 (0)	2 (2.6)	
Moderate	22 (28.6)	21 (27.6)	
Poor	52 (65.7)	52 (68.4)	
No evaluable (NE)	3 (3.9)	1 (1.3)	
Signet-ring cell carcinoma			0.469
Yes	29 (37.7)	34 (44.7)	
No	48 (62.3)	42 (55.3)	
Lauren type			0.431
Intestinal	28 (36.4)	30 (39.5)	
Mixed	17 (22.1)	23 (30.3)	
Diffused	17 (22.1)	14 (18.4)	
No evaluable (NE)^#^	15 (19.5)	9 (11.8)	
Tumor regression grade			0.269
Grade 0	12 (15.6)	6 (7.9)	
Grade 1	11 (14.3)	17 (22.4)	
Grade 2	46 (59.7)	42 (55.3)	
Grade 3	8 (10.4)	11 (14.5)	
ypT stage^*∗*^			0.915
T0	8 (10.4)	5 (6.6)	
T1a	8 (10.4)	5 (6.6)	
T1b	8 (10.4)	7 (9.2)	
T2	11 (14.3)	11 (14.5)	
T3	25 (32.5)	29 (38.2)	
T4a	16 (20.8)	17 (22.4)	
T4b	1 (1.3)	2 (2.6)	
ypN stage ^*∗*^			0.531
N0	36 (46.8)	30 (39.5)	
N1	15 (19.5)	13 (17.1)	
N2	12 (15.6)	11 (14.5)	
N3a	12 (15.6)	16 (21.1)	
N3b	2 (2.6)	6 (7.9)	
ypTNM stage^*∗*^			0.354
T0N0	7 (9.1)	5 (6.6)	
T0N1	1 (1.3)	0 (0.0)	
I	20 (26.0)	21 (27.6)	
II	24 (31.2)	16 (21.1)	
III	25 (32.5)	34 (44.7)	

LAG, laparoscopy-assisted gastrectomy; OG, open gastrectomy; NACT, neoadjuvant chemotherapy. #NE indicates that the Lauren classification was not evaluable since the tumor regression after NACT; ^*∗*^the yp stages were indicated by preoperative CT scan after neoadjuvant chemotherapy, and all staging was based on the eighth edition of the AJCC Cancer Staging Manual by the American Joint Committee on Cancer and International Union Against Cancer.

**Table 4:** Postoperative complications of population in our study.

Surgical and pathological features	LAG	OG	*p* value
	*N* = 77 (%)	*N* = 76 (%)	
Postoperative stays (days)	7.9 ± 2.1	9.3 ± 5.8	**0.009**
Postoperative complications			0.120
Yes	14 (18.2)	23 (30.3)	
No	63 (81.8)	53 (69.7)	
Clavien–Dindo classification^#^			
Grade I	1 (1.3)	1 (1.3)	>0.999
Pulmonary infection	1 (1.3)	1 (1.3)	>0.999
Grade II	12 (15.6)	18 (23.7)	0.207
Pulmonary infection	10 (13.0)	15 (19.7)	0.259
Gastroplegia	0 (0.0)	2 (2.6)	0.245
Digestive tract hemorrhage	0 (0.0)	1 (2.6)	0.497
Intraperitoneal hemorrhage	0 (0.0)	1 (1.3)	0.497
Lymphatic leakage	0 (0.0)	1 (1.3)	0.497
Pancreatic fistula	0 (0.0)	1 (1.3)	0.497
Arrhythmia	1 (1.3)	0 (0.0)	>0.999
Intestinal obstruction	2 (2.6)	0 (0.0)	0.497
Grade IIIa	0 (0.0)	4 (5.3)	0.058
Pulmonary infection	0 (0.0)	4 (5.3)	0.058
Grade IIIb	1 (1.3)	1 (1.3)	>0.999
Anastomotic leakage	0 (0.0)	1 (1.3)	0.497
Intestinal fistula	1 (1.3)	0 (0.0)	>0.999
Grade IV	0 (0.0)	0 (0.0)	NA
Grade V	0 (0.0)	0 (0.0)	NA
Unplanned readmission	1 (1.3)	5 (6.6)	0.116
Pulmonary infection	1 (1.3)	2 (2.6)	0.620
Intestinal obstruction	0 (0.0)	2 (2.6)	0.245
Gastroplegia	0 (0.0)	1 (1.3)	0.497

LAG, laparoscopy-assisted gastrectomy; OG, open gastrectomy; ^#^one patient can have more than 1 complication.

## Data Availability

Raw data of our study are available from the corresponding author of this study upon request.

## References

[1] Sung H., Ferlay J., Siegel R. L. (2021). Global cancer statistics 2020: GLOBOCAN estimates of incidence and mortality worldwide for 36 cancers in 185 countries. *CA: A Cancer Journal for Clinicians*.

[2] Wei W., Zeng H., Zheng R. (2020). Cancer registration in China and its role in cancer prevention and control. *The Lancet Oncology*.

[3] Van Cutsem E., Sagaert X., Topal B., Haustermans K., Prenen H. (2016). Gastric cancer. *The Lancet*.

[4] Cunningham D., Allum W. H., Stenning S. P. (2006). Perioperative chemotherapy versus surgery alone for resectable gastroesophageal cancer. *New England Journal of Medicine*.

[5] Ychou M., Boige V., Pignon J. P. (2011). Perioperative chemotherapy compared with surgery alone for resectable gastroesophageal adenocarcinoma: an FNCLCC and FFCD multicenter phase III trial. *Journal of Clinical Oncology*.

[6] Zhang X., Liang H., Li Z. (2021). Perioperative or postoperative adjuvant oxaliplatin with S-1 versus adjuvant oxaliplatin with capecitabine in patients with locally advanced gastric or gastro-oesophageal junction adenocarcinoma undergoing D2 gastrectomy (RESOLVE): an open-label, superiority and non-inferiority, phase 3 randomised controlled trial. *The Lancet Oncology*.

[7] Coccolini F., Nardi M., Montori G. (2018). Neoadjuvant chemotherapy in advanced gastric and esophago-gastric cancer. Meta-analysis of randomized trials. *International Journal of Surgery*.

[8] Japanese Gastric Cancer Association (2021). Japanese gastric cancer treatment guidelines 2018. *Gastric Cancer*.

[9] Kim H. H., Han S. U., Kim M. C. (2019). Effect of laparoscopic distal gastrectomy vs open distal gastrectomy on long-term survival among patients with stage I gastric cancer. *JAMA Oncology*.

[10] Inaki N., Etoh T., Ohyama T. (2015). A multi-institutional, prospective, phase II feasibility study of laparoscopy-assisted distal gastrectomy with D2 lymph node dissection for locally advanced gastric cancer (JLSSG0901). *World Journal of Surgery*.

[11] Hyung W. J., Yang H. K., Park Y. K. (2020). Long-term outcomes of laparoscopic distal gastrectomy for locally advanced gastric cancer: the KLASS-02-RCT randomized clinical trial. *Journal of Clinical Oncology*.

[12] Yu J., Huang C., Sun Y. (2019). Effect of laparoscopic vs open distal gastrectomy on 3-year disease-free survival in patients with locally advanced gastric cancer: the CLASS-01 randomized clinical trial. *JAMA*.

[13] Liu F., Huang C., Xu Z. (2020). Morbidity and mortality of laparoscopic vs open total gastrectomy for clinical stage I gastric cancer: the CLASS02 multicenter randomized clinical trial. *JAMA Oncology*.

[14] Zheng C., Xu Y., Zhao G. (2021). Outcomes of laparoscopic total gastrectomy combined with spleen-preserving hilar lymphadenectomy for locally advanced proximal gastric cancer: a nonrandomized clinical trial. *JAMA Network Open*.

[15] Li Z., Shan F., Ying X. (2019). Assessment of laparoscopic distal gastrectomy after neoadjuvant chemotherapy for locally advanced gastric cancer: a randomized clinical trial. *JAMA Surgery*.

[16] van der Wielen N., Straatman J., Daams F. (2021). Open versus minimally invasive total gastrectomy after neoadjuvant chemotherapy: results of a European randomized trial. *Gastric Cancer*.

[17] Li Z., Shan F., Wang Y. (2016). Laparoscopic versus open distal gastrectomy for locally advanced gastric cancer after neoadjuvant chemotherapy: safety and short-term oncologic results. *Surgical Endoscopy*.

[18] Xing J., Wang Y., Shan F. (2021). Comparison of totally laparoscopic and laparoscopic assisted gastrectomy after neoadjuvant chemotherapy in locally advanced gastric cancer. *European Journal of Surgical Oncology*.

[19] Khaled I., Priego P., Soliman H., Faisal M., Saad Ahmed I. (2021). Oncological outcomes of laparoscopic versus open gastrectomy after neoadjuvant chemotherapy for locally advanced gastric cancer: a retrospective multicenter study. *World Journal of Surgical Oncology*.

[20] Japanese Gastric Cancer Association (2011). Japanese classification of gastric carcinoma. *Gastric Cancer*.

[21] Ep Amin M. B., Edge S., Greene F. (2017). *The 8th Edition of the AJCC Cancer Staging Manual*.

[22] Liu K., Chen X. Z., Zhang W. H. (2019). Four-step procedure of laparoscopic exploration for gastric cancer in West China Hospital: a retrospective observational analysis from a high-volume institution in China. *Surgical Endoscopy*.

[23] Eisenhauer E. A., Therasse P., Bogaerts J. (2009). New response evaluation criteria in solid tumours: revised RECIST guideline (version 1.1). *European Journal of Cancer*.

[24] Japanese Gastric Cancer Association (2017). Japanese gastric cancer treatment guidelines 2014 (ver. 4). *Gastric Cancer*.

[25] Clavien P. A., Sanabria J. R., Strasberg S. M. (1992). Proposed classification of complications of surgery with examples of utility in cholecystectomy. *Surgery*.

[26] Dindo D., Demartines N., Clavien P. A. (2004). Classification of surgical complications: a new proposal with evaluation in a cohort of 6336 patients and results of a survey. *Annals of Surgery*.

[27] Hu Y., Huang C., Sun Y. (2016). Morbidity and mortality of laparoscopic versus open D2 distal gastrectomy for advanced gastric cancer: a randomized controlled trial. *Journal of Clinical Oncology*.

[28] Ajani J. A., D’Amico T. A., Almhanna K. (2016). Gastric cancer, version 3.2016, NCCN clinical practice guidelines in oncology. *Journal of the National Comprehensive Cancer Network*.

[29] Katai H., Sasako M., Fukuda H. (2010). Safety and feasibility of laparoscopy-assisted distal gastrectomy with suprapancreatic nodal dissection for clinical stage I gastric cancer: a multicenter phase II trial (JCOG 0703). *Gastric Cancer*.

[30] Hiki N., Katai H., Mizusawa J. (2018). Long-term outcomes of laparoscopy-assisted distal gastrectomy with suprapancreatic nodal dissection for clinical stage I gastric cancer: a multicenter phase II trial (JCOG0703). *Gastric Cancer*.

[31] Yang H., Zhang W. H., Ge R. (2021). Application of gross tissue response system in gastric cancer after neoadjuvant chemotherapy: a primary report of a prospective cohort study. *Frontiers Oncology*.

[32] Yang H., Zhang W. H., Liu K. (2021). Application of clockwise modularized laparoscopic lymphadenectomy in the suprapancreatic area, a propensity score matching study and comparison with open gastrectomy. *Surgical Endoscopy*.

